# Intraperitoneal injection of sodium pentobarbital has the potential to elicit pain in adult rats (*Rattus norvegicus*)

**DOI:** 10.1371/journal.pone.0238123

**Published:** 2020-09-03

**Authors:** J. N. Reimer, C. J. Schuster, C. G. Knight, D. S. J. Pang, V. S. Y. Leung

**Affiliations:** 1 Veterinary Clinical and Diagnostic Sciences, Faculty of Veterinary Medicine, University of Calgary, Calgary, AB, Canada; 2 Department of Clinical Sciences, Faculty of Veterinary Medicine, Université de Montréal, Saint-Hyacinthe, QC, Canada; 3 Groupe de Recherche de Pharmacologie Animale du Québec (GREPAQ), Faculty of Veterinary Medicine, Université de Montréal, Saint-Hyacinthe, QC, Canada; Universidade Federal do Rio de Janeiro, BRAZIL

## Abstract

An effective and pain-free killing method is required to achieve the goal of euthanasia, a “good death”. Overdose of sodium pentobarbital (PB) by intraperitoneal (IP) injection is a widely accepted technique in laboratory rats, but questions remain regarding pain associated with administration. As PB rapidly causes sedation and loss of consciousness, most studies have relied on indirect evidence of pain. The objective of this study was to assess pain associated with IP PB using an appropriate vehicle control. Adult male and female Sprague Dawley (SD) and female Wistar rats (N = 84) were block randomised by sex and strain to receive one of three treatments: 1) 800 mg/kg PB (pH 11), 2) saline or 3) vehicle controls (pH 11 or 12.5). Behavior (Rat Grimace Scale (RGS), writhing, back arching) was evaluated at baseline, before loss of righting reflex (LORR, PB group), and at 80s, 151s and 10 min post-injection (PI; saline and vehicle control groups). In the PB group, mean time to LORR was 78 ± 7.9 seconds. In the vehicle control groups, RGS scores were increased at 151s PI (SD: p = 0.0002, 95%CI 0.73 to 0.20) from baseline, as was relative frequency of writhing (SD: p < 0.0001; Wistar; p = 0.0004). RGS scores remained elevated 10 mins PI (SD: p = 0.0005, 95%CI 0.71 to 0.18; Wistar: p = 0.0234, 95%CI 0.91 to 0.07) but the relative frequency of writhing did not (p > 0.999). The RGS scores and the relative frequency of writhing remained low in the PB and saline groups (p > 0.05). These results show that, vehicle controls for IP PB result in signs associated with pain, pain may not be experienced following IP PB when LORR occurs quickly, and that the effects of PB limit behavioral pain assessments.

## Introduction

Approximately 9 million mice and rats are used in biomedical research in Canada and the European Union annually [1, 2]. As the majority of these animals are killed at project completion (or when a humane endpoint is reached), an effective, fast and pain-free killing method is essential.

Two methods for euthanasia are generally considered to be acceptable: 1) injection of barbiturates, such as sodium pentobarbital (PB), and 2) an overdose of an inhalant anesthetic [3, 4]. The use of inhalant anesthetics for euthanasia has been reported as being aversive to rodents [3, 5, 6]. Therefore, the intraperitoneal (IP) injection of an overdose of PB is widely considered to be a preferable method of euthanasia [3, 4]. The effect of IP PB has been reported to be rapid with loss of righting reflex (LORR) and cessation of heart beat (CHB) occurring around 111s and 283s, respectively, with 800 mg/kg PB [7]. However, it has been suggested that the highly alkaline pH of PB may cause pain upon IP injection [8] and current Canadian Council on Animal Care guidelines recommend the use of buffered, diluted PB, combined with a local anesthetic, such as lidocaine, to minimize this effect [3, 4]. Unfortunately, the guidelines do not provide specific recommendations on buffering, dilution or proportion of local anesthetic. As the pH of PB falls below 10, there is an increasing risk of drug precipitation, and this pH is still irritating to tissues [8, 9]. Furthermore, in titrating the addition of lidocaine to PB, precipitation has been reported to occur as the pH approaches 10, offering a potential explanation for why lidocaine added to PB reportedly reduces, but does not prevent, pain/nociception [8–10]. Few studies have explored the potential for pain or defined methods to assess pain associated with this killing method, to support the addition of local anesthetics to the injectate.

Studies that have explored pain associated with IP injection of PB report that while some behaviors associated with pain increase (e.g. writhing) others remain unchanged (e.g. the Rat Grimace Scale (RGS)) [9–11]. It is probable that the sedative-anesthetic and muscle relaxing properties of PB have the potential to interfere with behaviors used to evaluate pain, which may explain these conflicting results.

The aim of this study was to determine if the injection of a vehicle control (pH-matched to PB) is painful. It was hypothesized that pain behaviors would increase after the injection of a vehicle control but not following injection of PB.

## Methods

### Ethical statement

This study was approved by The Health Sciences Animal Care Committee at the University of Calgary (Animal Use Protocol AC11-0044) and was performed in accordance with the Canadian Council on Animal Care Euthanasia Guidelines (2010) and the Canadian Association of Laboratory Medicine (CALAM) Standards of Veterinary Care (2007).

### Experimental animals/housing and husbandry

Adult male (n = 33, 349g [201 to 435g] [median, range]) and female (n = 39, 267g [196 to 448g]) Sprague Dawley (SD) surplus rats, scheduled for euthanasia, were obtained from the University of Calgary Health Sciences Animal Resources Centre (HSARC). Female Wistar rats (n = 36, 239.5g [213 to 261g]) were obtained from Charles River Laboratories, Canada. Animals were housed in pairs in polycarbonate cages (47.6 x 26.0 x 20.3 cm, RC88D-UD, Alternate Design Mfg and Supply, Siloam Springs, Arizona, USA) with a bedding of wood shavings (Aspen chip, NEPCO, Warrensburd, NY, USA) and enrichments of a PVC tube, sizzle paper and nestlets. Rats were provided food (Prolab 2500 Rodent 5p14, Laboratory Animal Diet, LabDiet, St Louis, MO, USA*)* and tap water *ad libitum*. The housing environment consisted of a 12-hour light-dark cycle (light on at 7 am) and temperature and humidity of 23°C and 22%, respectively. Animals were checked twice daily and sentinel rats present in the housing room were negative for: rat parvoviruses, Toolan H1 virus, Kilham rat virus, rat minute virus and protoparvovirus NS-1, rat sialodacryoadenitis virus, rat theilovirus, *Pneumocystis carnii*, Sendai virus, pneumonia virus of mice, reovirus, *Mycoplasma pulmonis*, Lymphocytic choriomeningitis virus, adenovirus, hantavirus, *Encephalitozoon cuniculi*, cilia-associated respiratory bacillus, rat rotavirus, *Bordetella bronchiseptica*, *Corynebacterium kutscheri*, *Klebsiella oxytoca*, *Klebsiella pneumoniae*, *Rodentibacter pneumotropicus*, *Pseudomonas aeruginosa*, *Staphylococcus aureus*, *Streptococcus* β hemolytic and *Streptococcus pneumoniae*, *Proteus mirabilis*, *Salmonella* and other bacteria, and endo- and ectoparasites.

Animals were block randomised (list randomizer, random.org) by sex and strain to receive one of three treatments: 1) 800 mg/kg pentobarbital (PB, Euthanyl, 240 mg/mL, Bimeda-MTC Animal Health Inc., Cambridge, ON, Canada, pH of 11.018 ± 0.009 upon testing); 2) Saline controls at 3.33 mL/kg (Sodium Chloride 0.9% Injection, pH 5.3 (4.5–7.0), FK Std., Fresenius Kabi Canada, Mississauga, ON, Canada; volume equal to PB) and 3) vehicle controls (SD: vehicle control pH of 11.0 at 3.33 mL/kg. Wistars: vehicle control pH of 12.5 at 3.33 mL/kg). The Wistar vehicle treatment group was included to provide a comparison with a higher pH previously reported for a PB solution used in this strain [8]. The saline control pH falls in a range not associated with pain [12, 13]. Vehicle controls were prepared as: propylene glycol (40%), ethanol (10%), water for injection and pH balanced to pH 11 or 12.5 (Chief Pharmacy, Calgary, AB, CAN and Chiron Compounding Pharmacy Inc., Guelph, ONT, CAN). Each injection was prepared in a 3 mL syringe with a 25-gauge 5/8” needle and 0.01 mL of blue food colouring added (Blue Food Colour, Club House, McCormick Canada, London, Canada). Rats were excluded and replaced if misinjection was confirmed at necropsy. Both experimenters (JR, CS) were blinded to the treatments and all assessments were performed between 7 am and 6 pm.

### Video recording (for behavioral assessments)

Three days before the experimental day, all rats were habituated daily to handling by the experimenters and placement in the observation chamber (14 x 27 x 21 cm) for 10 minutes. During handling, both experimenters habituated the rat to the two-person injection technique: one experimenter cradled the rat in a backpack hold in dorsal recumbency with a 30° head down angle and extended the left pelvic limb. The other experimenter extended the right pelvic limb while simultaneously holding a capped hypodermic needle against the abdominal wall of the right caudal quadrant at a 45° angle to the body wall, as previously described [7].

On the testing day, rats were weighed before placement into the observation box for baseline recording (Panasonic HC-V720P/PC, Panasonic Canada Inc., Mississauga, ON, Canada) for 10 minutes. Rats were then removed from the box and given a single intraperitoneal (IP) injection using the two-person injection technique. After injection (INJ), rats were immediately returned to the observation chamber for observation and video-recording until loss of righting reflex (LORR) occurred or until 10 minutes elapsed (whichever came first). At the first signs of ataxia, LORR was assessed by attempting to place the rat in left lateral recumbency, followed by dorsal recumbency. If the rat remained on its back for 10 seconds LORR was considered to be achieved. If the rat righted itself, LORR was reassessed every 30 seconds until achieved or until 10 minutes had elapsed. Following LORR, the animal was monitored for cessation of breathing (CB, visual assessment). At CB, the animal was placed in left lateral recumbency and monitored for cessation of heartbeat (CHB, thoracic auscultation with stethoscope). If CHB did not occur within 20 minutes of injection, the rat was euthanized with an overdose of inhaled isoflurane (IsoFlo®, Abbot Animal Health, Abbott Laboratories, North Chicago, IL, USA). Times to achieve LORR, CB and CHB were recorded for the PB group only.

## Behavioral assessments

Image collection for RGS scoring and behavioral assessments were performed by observers (JR,VL) blinded to treatment group. Both observers completed structured training in applying the RGS [14]. For RGS scoring, three images were selected when the rats were not performing behaviors that could influence facial expressions (i.e. sleeping, sniffing, eating and grooming). Each observation interval was divided into three approximately equal periods and a single image from each period was selected. Image selection was based on image quality (clarity of facial features) and frontal facing images. Three images were collected during the 10 minute baseline video for each animal. For post-injection (PI) videos of animals that had LORR, three images were selected before LORR occurred (duration ranged from 56 to 105s in the data collected). For PI videos in which LORR did not occur, three images were selected from each of the following intervals: first 80s of the video (mean time to achieve LORR in this study), from a 30s segment of the video (121 to 151s after IP injection, based on data showing LORR may not be achieved for up to 151s) [7] and during the last minute of observation (9 to 10 minutes after IP injection). Collected images were inserted into commercial presentation software (Microsoft PowerPoint, version 15.0, Microsoft Corporation, Redmond, WA, USA) and randomized with a macro (http://www.tushar-mehta.com/powerpoint/randomslideshow/index.htm). Each image was assessed as previously described [15]. Briefly, four action units (orbital tightening, ear changes, nose/cheek flattening and whisker changes) were scored from 0 to 2 (increasing score represents increasing pain). Additionally, the following behaviors were assessed as relative frequencies: writhing and back arching [16]. These behaviors were assessed over the first 151s of both BL and PI videos (or before LORR). In addition, writhing was also assessed before LORR or during the first 80s PI and during the last minute (9–10 minutes PI, where LORR did not occur). Writhing was defined as the contraction of the lateral abdominal walls where the abdomen appears concave. Back arching was defined as the arching of the back (with the abdomen pushed towards the ground or a vertical upwards arch).

### Necropsy

Skin was incised along the ventral midline from the sternum to pubis and reflected back using blunt dissection. The linea alba was incised and the muscles along the costal arch were cut to expose the peritoneal cavity, which was photographed with viscera in place. The gastrointestinal tract from cardia to descending colon was then removed and any sections with blue staining opened to determine if staining was serosal or intraluminal. The liver, abdominal wall surrounding the injection site, and excised gastrointestinal tract were placed in 10% neutral buffered formalin solution for fixation of at least 7 days.

### Histological analysis

For histologic analysis, formalin-fixed samples of liver, gastrointestinal tract and abdominal wall muscle were embedded in paraffin, sectioned at 4 micrometer thickness and stained routinely with haematoxylin and eosin (H&E) stain. Samples were not collected from animals in which a misinjection was identified based on initial gross evaluation of abdominal contents. Slides were evaluated by a US board-certified veterinary pathologist (CGK) blinded to treatment for evidence of mesothelial (peritoneal) and submesothelial reaction, damage or inflammation.

### Statistical analysis

Data were analysed with commercial statistical software (Prism 6.07, GraphPad Software, La Jolla, CA, USA). Normality was assessed with the D’Agostino-Pearson omnibus normality test. Physiologic data and RGS scores approximated a normal distribution while the relative frequency of writhing and back arching did not. Differences from baseline for RGS data were assessed with a paired t-test (for PB data) or one-way ANOVA and post-hoc Dunnett’s (for saline and vehicle control data). Differences from baseline for writhing and back arching were assessed with a Wilcoxon test (for PB data (writhing and back arching), saline and vehicle control (back arching)) or a Friedman’s test and post-hoc Dunn’s (saline and vehicle control data (writhing)). Differences between saline and vehicle control data were assessed with a two-way ANOVA and post-hoc Bonferroni’s test. Post-hoc tests for within and between group analysis were only performed if there was a significant interaction or main effect. From the PB group, the following time periods are presented with descriptive statistics: injection–LORR, LORR–CB, CB–CHB, injection–CHB. Strains were analysed separately because of the different pH levels of the vehicle controls. Sample sizes were estimated (G*Power 3.1.9.2, Germany) for the two main behavioral outcomes: RGS and writhing. For the RGS, a sample size of 12 animals per group was estimated based on: alpha = 0.05, power = 0.8, SD = 0.25 and expected mean difference of 0.3 [14]. For writhing, a sample size of 12 animals per group was estimated based on: alpha = 0.05, power = 0.8, SD = 4.74 and expected difference of 6 [15]. A p-value of < 0.05 was considered statistically significant for all comparisons and 95% confidence intervals for the mean/median difference presented where available. Data in the figures are presented as mean ± SEM (RGS) or as median ± IQR (relative frequency of back arch and writhing). Data in text are presented as mean ± standard deviation. Data supporting the results are available in an electronic repository: https://doi.org/10.7910/DVN/9OUVJ4

## Results

The misinjection rate was 22.2% (24/109), similar to previous studies [7, 17–19]. These rats were excluded from analysis. An additional rat was excluded due to a dosing error. The total number of SD females, SD males and Wistar females were n = 30 (254g [196 – 440g], 11–15 weeks old), n = 27 (346 g [201 – 425g], 8–13 weeks old) and n = 27 (240g [213 – 261g], 9–12 weeks old), respectively. As block randomization was maintained, these animals were divided equally into the three treatment groups: SD females (n = 10), SD males (n = 9) and Wistar females (n = 9). A maximum of 702 images could be captured for RGS assessment. The successful image capture rate was 99.6% with only three images that could not be captured from two rats because they were grooming during the majority of the observation period. Tissue samples were collected from 74 rats. Due to a planning error, formalin was unavailable for sample storage in 23 rats. Tissue samples were not taken from 18 rats as misinjection was identified at initial necropsy. Tissue samples from 6 rats were excluded because of misinjections identified during histological examination. There were 22 samples from SD males (PB: n = 4, saline: n = 7, vehicle: n = 11), 24 from SD females (PB: n = 7, saline: n = 9, vehicle: n = 8) and 21 from Wistar female rats (PB: n = 3, saline: n = 9, vehicle: n = 9).

### RGS

**Fig 1:**
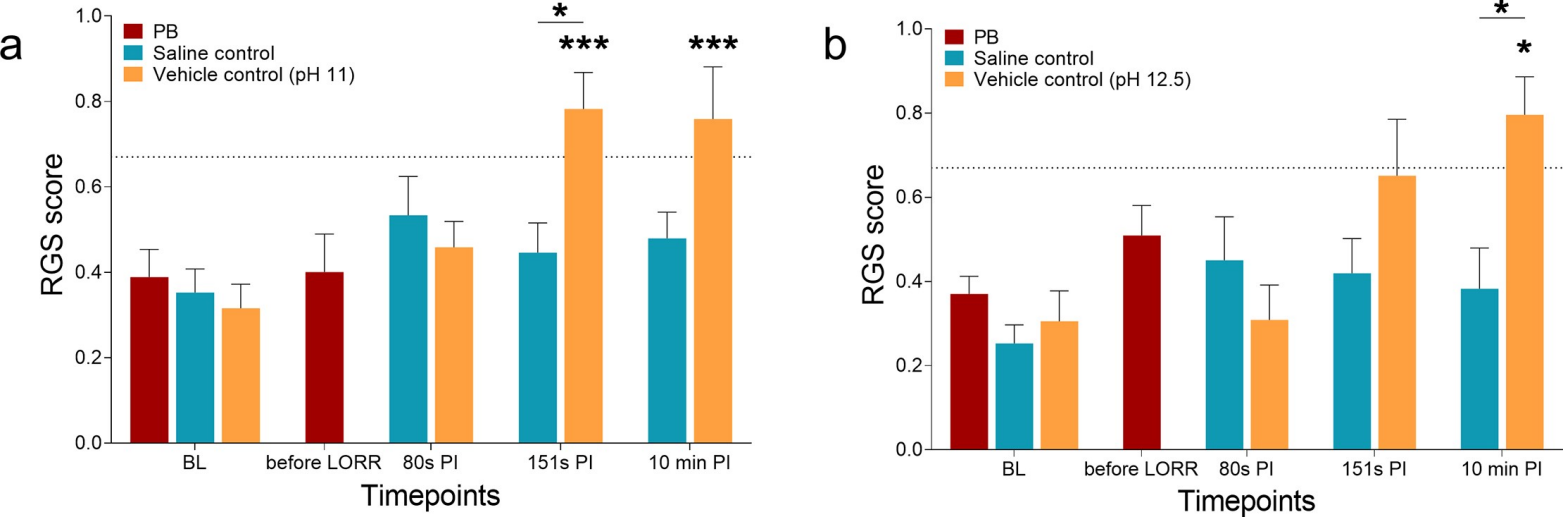
RGS scores of rats that received sodium pentobarbital (PB), saline controls or vehicle controls (pH 11 or 12.5). (a) In the female and male Sprague Dawley group, significant increases from baseline were observed in the vehicle control group at the 151s and 10 min post-injection (PI) timepoints (p < 0.01). Differences between saline and vehicle control groups were observed at the 151s PI timepoint (p < 0.05). (b) In the female Wistar group, a significant increase from baseline was only observed at 10 min PI timepoint in the vehicle control group (p < 0.05). Differences between groups were observed at the 10 min PI timepoint (p < 0.05). The horizontal dotted line represents a previously established intervention threshold of 0.67. [19] Data presented as mean ± SEM. *p < 0.05, **p < 0.01, ***p < 0.001.

Increases in RGS scores were only observed in rats that received the vehicle controls (SD: F (3, 54) = 8.8, p < 0.0001; Wistar: F (1.98, 15.9) = 6.4, p = 0.009; Fig 1A and 1B). There were increases in RGS scores from BL after 10 min PI (SD: p = 0.0005, 95% CI -0.71 to -0.18; Wistar: p = 0.024, 95% CI -0.91 to -0.074). Increases from BL at 151s PI were also observed in the SD rats (p = 0.0002, 95% CI -0.73 to -0.20) but not in the Wistar rats (p = 0.188, 95% CI -0.85 to 0.16). The mean of these scores crossed a previously established analgesic intervention threshold of 0.67 [19]. The RGS scores at 80s remained similar to BL (SD: p = 0.427, 95% CI -0.41 to 0.12; Wistar: p > 0.999, 95% CI -0.23 to 0.23). At timepoints when RGS scores were high (151s and 10 min PI in the vehicle control groups), the number of rats that displayed RGS scores exceeding the intervention threshold [19] was highest (Table 1).

**Table 1:** Number of rats that had an RGS score greater than a previously established threshold (> 0.67) after treatment with sodium pentobarbital (PB), saline control or vehicle controls (pH 11 or 12.5).

Time points	Treatment groups					
	PB		Saline control		Vehicle control	
	n	%	n	%	n	%
SD rats						
BL	4	21.1	2	10.5	2	10.5
before LORR	4	21.1	-	-	-	-
80s PI	-	-	7	36.8	3	15.8
151s PI	-	-	4	21.1	10	52.6
10 min PI	-	-	2	10.5	8	42.1
Wistar rats						
BL	0	0.0	0	0.0	0	0.0
before LORR	2	22.2	-	-	-	-
80s PI	-	-	1	11.1	1	11.1
151s PI	-	-	1	11.1	4	44.4
10 min PI	-	-	1	11.1	5	55.6

Comparisons between saline and vehicle controls groups showed a significant interaction effect (SD: F (3, 108) = 4.86, p = 0.003; Wistar: F (3, 48) = 3.70, p = 0.02; Fig 1A and 1B). Significant increases in RGS scores occurred between saline and vehicle controls at 151s (SD: p = 0.018, 95% CI -0.63 to -0.05) and 10 min (Wistar: p = 0.03, 95% CI -0.79 to -0.04). RGS scores were similar at BL (SD: p > 0.999, 95% CI -0.17 to 0.25; Wistar p > 0.999, 95% CI -0.30 to 0.19), 80s (SD: p > 0.999, 95% CI -0.21 to 0.37; Wistar: p > 0.999, 95% CI -0.23 to 0.52), 151s (Wistar: p = 0.66 95% CI -0.69 to 0.22) and 10 min (SD: p = 0.21 95% CI -0.65 to 0.087).

The total number of SD and Wistar rats were 19 and 9 per treatment group, respectively. n = number of rats that had a RGS score greater than a previously established intervention threshold (> 0.67). [20]% = percentage of rats that displayed a RGS score greater than >0.67. BL, baseline. LORR, loss of righting reflex. PI, post-injection.

The PI RGS scores of animals which received PB (SD: p = 0.876, 95% CI -0.14 to 0.17; Wistar: p = 0.100, 95% CI -0.034 to 0.31) or saline (SD: F (3, 54) = 1.8, p = 0.160; Wistar: F (3, 24) = 1.30, p = 0.300) displayed scores similar to BL.

### Writhing

**Fig 2:**
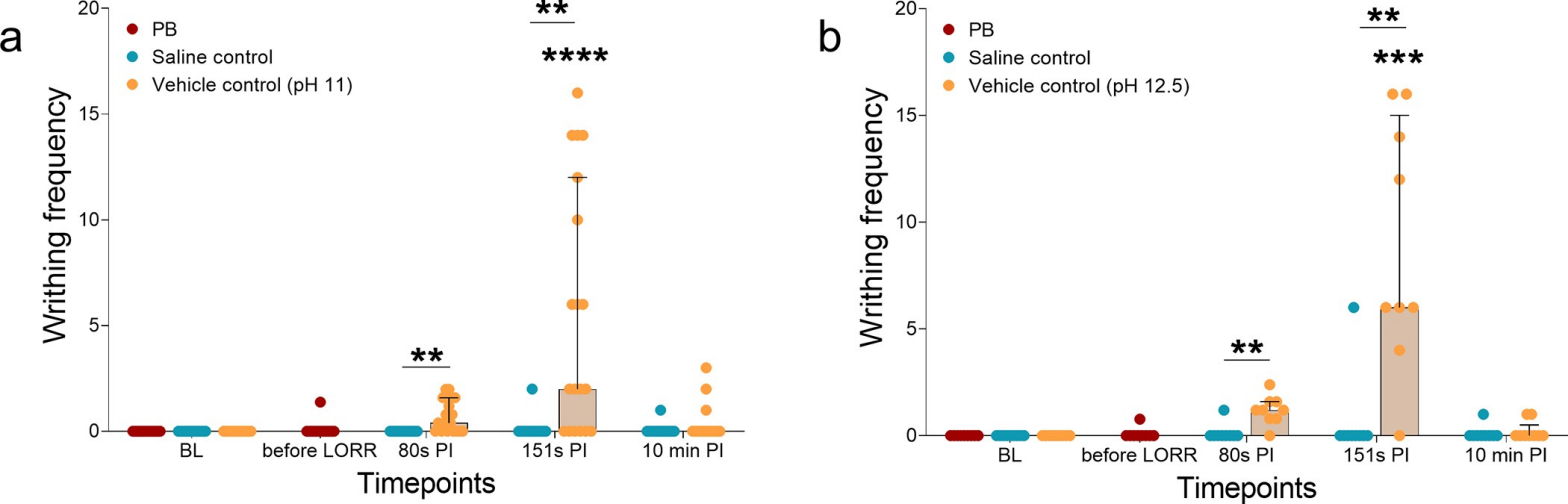
The relative frequency of writhing displayed by rats treated with sodium pentobarbital (PB), saline controls or vehicle controls (pH 11 or 12.5). Significant increases from baseline (BL) were only observed at the 151s post-injection (PI) timepoint in both female and male Sprague Dawley rats (p < 0.0001, a) and female Wistar rats (p < 0.001, b). Increased writhing occurred between saline and vehicle control groups at the 80s and 151s PI timepoints in both female and male Sprague Dawley and Wistar rats (p < 0.01). Data presented as median ± IQR. **p < 0.01, ***p < 0.001, ****p < 0.0001.

Similar to RGS scores, increases in the relative frequency and number of animals that displayed writhing behaviors were observed in animals that received vehicle controls (SD and Wistar: p < 0.0001; Fig 2A and 2B, Table 2). A higher relative frequency of writhing was observed at 151s PI compared to BL (SD: p < 0.00001; Wistar; p = 0.0004). At 80s (SD: p = 0.114; Wistar: p = 0.085) and 10 min PI (SD and Wistar: p > 0.999) the relative frequency of writhing was similar to BL. In PB and saline treatment groups, there was no overall effect of treatment within groups (PB; SD: p > 0.999; Wistar: p > 0.999. Saline controls; SD: p = 0.392; Wistar: p > 0.999, Fig 2A and 2B). No rats displayed writhing behaviour during baseline (Table 2). The greatest number of rats that displayed writhing behaviour was observed in the vehicle control groups at 80s and 151s PI.

**Table 2:** Number of rats that displayed writhing behaviour at least once after treatment with sodium pentobarbital (PB), saline controls or vehicle controls (pH 11 or 12.5).

Timepoints	Treatment groups					
	PB		Saline control		Vehicle control	
SD rats						
	n	%	n	%	n	%
BL	0	0.0	0	0.0	0	0.0
before LORR	1	5.3	-	-	-	-
80s PI	-	-	0	0.0	12	63.2
151s PI	-	-	1	5.3	13	68.4
10 min PI	-	-	1	5.3	3	15.8
Wistar rats						
BL	0	0.0	0	0.0	0	0.0
before LORR	1	11.1	-	-	-	-
80s PI	-	-	1	11.1	8	88.9
151s PI	-	-	1	11.1	8	88.9
10 min PI	-	-	1	11.1	2	22.2

The total number of SD and Wistar rats were 19 and 9 per treatment group, respectively. n = number of rats that displayed writhing behaviour. % = percentage of rats that displayed writhing behaviour. BL, baseline. LORR, loss of righting reflex. PI, post-injection.

Comparisons between saline and vehicle controls revealed a significant interaction effect (SD: F (3, 108) = 16.1, p < 0.0001; Wistar: F (3, 48) = 16.0, p < 0.0001). Significantly increased writhing was observed at 80s (SD: p = 0.002, 95% CI 1.18 to 0.245; Wistars: p = 0.005, 95% CI 1.80 to 0.32) and 151s (SD: p = 0.003, 95% CI 9.22 to 1.73; Wistars: p = 0.10, 95% CI 14.4 to 2.0). No differences were observed at BL (SD and Wistars: p = 1.00, 95% CI 0.0 to 0.0) and 10 min (SD: p = 0.77, 95% CI -0.80 to 0.27; Wistars: p > 0.999, 95% CI -0.63 to 0.41).

Upon visual inspection of the data, a sex effect was apparent: SD females in the vehicle control groups (S1A Fig) displayed a higher relative frequency of writhing behavior at 151s PI in comparison to the male SD rats (S1B Fig). There was an overall significant effect in both females (p < 0.0001) and males (p = 0.011). Significant increases from baseline at 151s were observed in female (p < 0.0001) but not male rats (p = 0.25). The relative frequency of writhing was similar to baseline at all other timepoints in both SD males and females that received the vehicle controls (at 80s PI; males: p > 0.999, females: p = 0.113. at 10 min PI; males: p > 0.999, females: p = 0.896). Of rats that received saline, there were no differences from baseline in the SD males (80s, 151s and 10 min PI: p > 0.999). There were no significant differences from BL in writhing behavior in the PB group (PB; female: p > 0.999; male: p = 1.00).

Comparisons between saline and vehicle controls showed a significant interaction effect in SD female (F (3, 54) = 25.3, p < 0.0001) and male rats (F (3, 48) = 4.01, p = 0.013) (S1A and S1B Fig). Significant increases in writhing were observed at 151s (females: p < 0.0001 95% CI -11.5 to -6.86; males: p = 0.0009 95% CI 2.21 to 0.46). There were no differences at BL (females: p > 0.999 95% CI -2.34 to 2.34; males: p > 0.999 95% CI -0.87 to 0.87), 80s (p = 0.84 95% CI -3.49 to 1.18; males: p > 0.999 95% CI -1.09 to 0.65) and 10 min (p > 0.999 95% CI -2.84 to 1.84; males: p > 0.999 95% CI -0.87 to 0.87).

### Back arching

**Fig 3:**
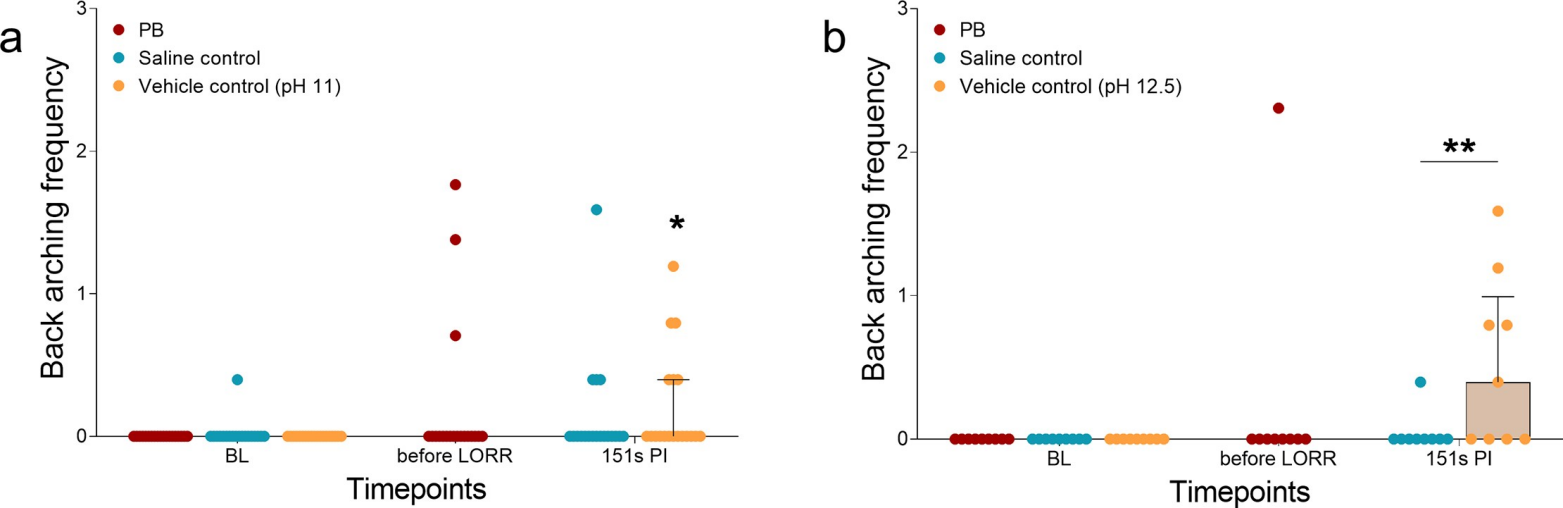
The relative frequency of back arching displayed by rats treated with sodium pentobarbital (PB), saline control or vehicle controls (pH 11 or 12.5). (a) With the female and male Sprague Dawley rats, significant increases from baseline (BL) were only observed in the vehicle control group at the 151s PI timepoint (p < 0.05). Differences between saline and vehicle control groups were not observed (p > 0.05). (b) With female Wistar rats, there were no significant differences between BL and the different timepoints (p > 0.05). A significant increase in back arching occurred between saline and vehicle control groups at 151s PI (p < 0.01). Data presented as median ± IQR. *p < 0.05, **p < 0.01.

SD rats in the vehicle control group expressed back arching more frequently during the 151s PI period than at BL (vehicle control: p = 0.031; Fig 3A). This increased frequency was not observed in Wistar rats (vehicle control: p = 0.063; Fig 3B). For both SD and Wistar rats that received PB or saline, the relative frequency of back arching was similar to baseline (PB: SD; p = 0.250, Wistar; p > 0.999. Saline: SD; p = 0.313, Wistar: p > 0.999). Back arching behaviour was observed during the PI timepoints but was never observed during BL (Table 3).

**Table 3:** Number of rats that displayed back arching behaviour at least once after treatment with sodium pentobarbital (PB), PB with lidocaine (PB+lido), saline controls or vehicle controls (pH 11 or 12.5).

	Treatment groups					
Timepoints	PB		Saline control		Vehicle control	
	n	%	n	%	n	%
SD rats						
BL	0	0.0	0	0.0	0	0.0
before LORR	3	15.8	-	-	-	-
151s PI	-		4	21.1	6	31.6
Wistar rats						
BL	0	0.0	0	0.0	0	0.0
before LORR	1	11.1	-	-	-	-
151s PI	-	-	1	11.1	5	55.6

The total number of SD and Wistar rats were 19 and 9 per treatment group, respectively. n = number of rats that displayed back arching behaviour. % = percentage of rats that displayed back arching behaviour. BL, baseline. LORR, loss of righting reflex. PI, post-injection.

Comparisons between saline and vehicle controls showed a significant interaction effect (F (1, 16) = 5.69, p = 0.03) in female Wistar rats, with a significant increase in back arching in the vehicle control group at 151 s PI (p = 0.004, 95% CI 0.82 to 0.15, Fig 3B). No difference was observed between vehicle and saline control groups at BL (p > 0.999, 95% CI -0.34 to 0.34). In male and female SD rats, there was a significant time effect (F (1, 36) = 7.38, p = 0.01); however, there were no significant differences between groups at BL (p > 0.999, 95% CI -0.18 to 0.22) and 151 s (p = 0.94, 95% CI -0.26 to 0.13).

Visual inspection of the data for sex differences in SD rats was also performed and no differences were apparent in both SD female and male rats (S2A and S2B Fig). Comparisons to BL did not reveal significant increases when the sexes were separated (PB; SD females: p = 0.500, SD males: p = 0.999. Saline; SD females: p > 0.999, SD males p = 0.500. Vehicle control; SD females: p = 0.125, SD males: p = 0.500).

Comparisons between saline and vehicle controls revealed a significant time effect for SD female (F (1, 18) = 5.83, p = 0.03) but not male rats (F (1, 16) = 2.45, p = 0.14). In females, increased back arching occurred at 151s PI (p = 0.02, 95% CI 0.52 to 0.03), with no difference at BL (p > 0.999, 95% CI -0.25 to 0.25).

### Physiologic data

The time period data for the PB group was as follows: INJ to LORR; 78 ± 7.9 seconds, LORR to CB; 90 ± 19 seconds, CB to CHB; 84 ± 16 seconds, INJ to CHB; 252 ± 24 seconds. Eleven of the thirteen rats that received a misinjection of PB achieved LORR at 105.1 ± 39.3s (range 76 to 203s). The other two rats never achieved LORR.

## Histologic analysis

Each of the 74 slides included up to 6 representative sections of small and large intestine (3 gut sections: n = 8; 4 gut sections: n = 19; 5 gut sections: n = 29; 6 gut sections: n = 12). The majority of slides included a section of liver (n = 67). Twenty-nine included a section of abdominal wall and 8 included a section of pancreas attached to the associated duodenal segment. No evidence of mesothelial or submesothelial reaction, damage or inflammation was seen in any section aside from rare foci of mechanical trauma caused by the injection needle.

## Discussion

The results of this study show that behaviors associated with pain are present after vehicle injection but not after PB injection. When IP PB injection goes smoothly (no misinjection) and loss of the righting reflex occurs within 80 seconds, it appears that pain (as indicated by the behaviors measured) does not occur. However, at the dose of PB studied, there remains an inherent individual variability in time from injection to LORR. The mean ±2SD time for this period was 93.8 seconds in this study and 151.0 seconds in a previous study [7]. Therefore, when time to LORR exceeds 80 seconds, there is a possibility of pain occurring. An underlying assumption in this study was that if rats receiving PB did not exhibit pain behaviors, this was due to an inability to express these behaviors (reflecting the sedation and anesthesia generated by PB). Converging evidence from electrophysiology or functional imaging studies are needed to explore evidence of pain pathway activation following IP PB [21]. Importantly, the minimal expression of pain behaviors in the PB group should not be interpreted as analgesia from PB. Pentobarbital, like other barbiturates, produces dose dependent sedation and general anesthesia (and eventually death, as the dose is increased), mediated through the GABA_A_ receptor [22]. Barbiturates do not have analgesic properties so that failure to respond to a potentially noxious stimulus reflects sedation or anesthesia and should not be interpreted as an analgesic effect [23, 24].

As outlined in the CCAC [3] and AVMA [4] euthanasia guidelines, during the euthanasia of animals, distress and pain must be minimized. The use of barbiturates, such as sodium pentobarbital, is designated as an acceptable method and preferred over other methods, such as inhalant anesthetics, because they are fast acting, inexpensive, readily available, have a long shelf life and are supposedly less aversive [4]. However, the methods to assess pain associated with IP PB have not been well defined [4].

The highly alkaline pH of PB solution (typically pH 11–12) has been suggested as a cause of pain when delivered IP [3, 8]. Several studies have reported that pain is present during IP PB injection because of changes in behaviors (i.e. increased writhing, reduction of locomotion and rearing, directed grooming) [8, 9, 11, 25], altered levels of molecular markers (i.e. increase in spinal c-fos [10]) and the appearance of redness in the peritoneal cavity, indicative of inflammation [11]. Studies have also reported that writhing and spinal c-fos levels decreased when a local anesthetic, such as lidocaine or bupivacaine, was administered [9–11]. These results all suggest that pain may be associated with IP PB injections. Unfortunately, not all of these studies have undergone peer review [8, 11]. Interpretation of changes in molecular markers alone is challenging as expression is altered by neuronal activation that may not be specific to nociception, and nociception is not necessarily indicative of pain [10, 26]. Furthermore, short periods of noxious stimuli, as seen during successful IP PB injection, are difficult to identify using changes in expression of many molecular markers [26]. Additionally, a failure to document a change in motor behavior in the presence of a drug causing sedation and reduced motor function, such as PB, should be interpreted cautiously. This could explain the apparent failure of the RGS to change following IP PB in one study and is supported by the results presented here [9].

Several behaviors have been used to study abdominal pain (often following laparotomy) [15, 16, 27]. Of these, the grimace scales and writhing behavior were selected as they have also been shown to increase with exposure to a noxious substance injected IP and to decrease with analgesic use [27, 28]. Back arching was reported to increase after laparotomy [16] but has not been specifically reported to increase in response to the IP injection of noxious substances in rodents. These behaviors did not change consistently in this study.

### Writhing behavior

An increase in writhing behavior was observed only in the vehicle control groups at 151s PI and not in the PB groups. This differs from previous studies that reported an increase in writhing duration after IP injection of PB and the presence, though at a low incidence, of writhing after IP PB [7, 9]. These differences may result from the different methods of assessing writhing (relative frequency vs duration vs presence/absence). We elected to use relative frequency to account for the different observation periods between individuals and treatment groups. Zatroch et al. [7] reported writhing in fewer than 50% of rats injected with either 200 or 800 mg/kg PB and observed writhing in a small number of rats (n = 2/9) receiving a saline control injection, highlighting the importance of this control. Interestingly, in the study reported here, writhing behavior was not sustained at the 10 minute observation period. This could reflect a reduction in pain over time or perhaps an increase in pain to a level that inhibited further writhing.

An unexpected effect of sex was observed, with male SD rats displaying fewer bouts of writhing than females. The mechanism of this difference is unknown. However, this effect of sex was not maintained with the RGS scores, suggesting that other factors may have been involved.

### RGS

Similar to writhing behaviors, RGS scores only increased significantly in the vehicle control groups. In contrast to the writhing data, the RGS scores were increased at both 151s and 10 minute time points, and the average scores were close to or exceeded a previously established threshold associated with pain [20]. The maintenance of low RGS scores in the PB treated animals and increase in scores in the vehicle control group highlights the potential for agents with sedative/anesthetic properties to mask behavioral expression. This is a likely explanation for the failure of RGS scores to change in the study of Khoo et al. [9]

The results from both writhing and RGS observations indicate that an IP injection of PB is painful due to the alkaline pH of the injectate. The low relative frequency of writhing and the low RGS scores in the PB groups support our hypothesis that the sedative effect of PB inhibits behavioral expression. Importantly, neither writhing nor RGS scores were increased at the 80s time point, suggesting that the onset of pain occurs after this time. Therefore, as discussed above, if loss of consciousness occurs rapidly, it is possible that pain may not be experienced following IP PB. This has important implications for situations in which time to unconsciousness is delayed, such as with misinjection and with lower doses of PB [7]. Two previous studies have shown that lower doses of IP PB delays LORR and loss of consciousness: animals that received IP PB at a dose of 200 mg/kg took on average 25% longer to achieve LORR than those given 800 mg/kg, and animals that received IP PB at a dose of 150 mg/kg took a longer time to lose consciousness (212 ± 10.5 seconds; using loss of muscle tone as a proxy for loss of consciousness) than the results reported here [7, 8]. Our study also showed that rats receiving a misinjection of PB take longer to achieve LORR. Unfortunately, it appears that misinjections are always possible, in part due to the variable location of the cecum [7, 18, 19, 29, 30].

The descriptive data from the PB group are broadly similar to previous work, in which the same time periods were studied [7]. In the study reported here, time from INJ-LORR was approximately 30% faster, while LORR-CB took approximately 37% longer, so that the overall time from INJ-CHB was similar.

## Histology

Histologic examination of the serosal surfaces of abdominal organs harvested from rats euthanized by IP injection does not offer information about pain associated with the procedure. Organs were evaluated for evidence of acute inflammation, including serosal and subserosal vasodilation and vascular congestion, neutrophilic margination and transmigration, mesothelial cell swelling, or mesothelial cell necrosis and sloughing. None of these features were seen and no treatment group differences were detected. This was not unexpected, as most histologic changes associated with acute inflammation take longer than 20 minutes to develop, which was the maximum interval between injection and death in this trial. The exception to this is vasodilation / active hyperemia, which can occur seconds to minutes after injury. Grossly visible peritoneal and serosal reddening has been previously described after IP PB injection [10, 24], but this was not seen in this study. Histologically, it is difficult to attribute significance to the presence or absence of red blood cells in blood vessels for several reasons. While the heart is still active blood may redistribute around the time of death through dysregulated autonomic control of vessels. After death, blood may redistribute under the influence of gravity. Blood can also be lost from vessels during tissue trimming and histologic processing. Finally, physiologic hyperemia due to digestion and peristaltic activity may cause intestinal sections to appear congested; this should not be interpreted as peracute inflammation. Therefore, although gross reddening of serosal surfaces caused by IP PB injection cannot be ruled out, it was not seen in this trial and was not supported histologically.

## Limitations and future studies

This study only used one dose of PB. This dose was selected from previous work demonstrating benefits in terms of a faster death and reduced variability of effects [7, 18, 19, 29]. The occurrence of misinjections appears to be an inherent drawback of IP injections, leading to the ongoing search for alternative injectable euthanasia methods [31–33].

In creating the vehicle control, the authors recognise that several ingredients were used. An assumption was made that pH is the determining factor in eliciting pain; however, the study design did not allow us to rule out that one or more of the ingredients may have resulted in pain. Of these, propylene glycol is reported as well-tolerated [31, 34]. Ethanol is not inert and has been tested previously as a euthanasia agent in mice and rats [31]. In rats, no signs of distress or abdominal pain were observed when using an ethanol dose ranging from 9.2–20.1 g/kg, though at these high doses most rats displayed ataxia, which may have interfered with displaying or interpreting behavioral signs of pain. In mice, a significant increase in Mouse Grimace Scale scores occurred following the higher dose of ethanol (15.3 g/kg), with no concurrent increase in signs of abdominal pain (abdominal constriction, hunched posture) [31].

The analgesic intervention threshold was applied here to aid interpretation of the changes in RGS scores. It is important to note that the original derivation of the threshold was in adult female SD rats that underwent surgery involving the skin and muscular systems. However, recent work showed that the RGS and intervention threshold identified the onset of acute and chronic colitis (a model of visceral pain) in male and female SD rats [20, 35]. The RGS and intervention threshold has also been successfully applied to distinguish analgesic effects in female Wistar rats (laparotomy model, analgesic testing) [36, 37].

In conclusion, the vehicle used to formulate sodium pentobarbital causes pain in adult male and female SD rats and female Wistar rats. The relevance of this result is strongly dependent on the time to achieve loss of righting reflex; when this occurs quickly, in under 80s, pain may not be experienced. These results highlight the importance of including a vehicle control group in studies and the limitations of interpreting behaviors in the presence of an agent with sedative-anesthetic properties, such as PB.

## Supporting information

S1 Fig The writhing frequency of male and female Sprague Dawley rats that received sodium pentobarbital (PB), saline controls or vehicle controls (pH 11). (a) In the female rat group, significant increases from baseline were observed from the vehicle control group at the 151s post-injection (PI) timepoint (p < 0.0001). Differences between saline and vehicle control groups were also observed at the 151s PI timepoint (p < 0.0001). (b) In the male rat group, differences between saline and vehicle control group were observed at the 151s PI timepoint (p < 0.001). Data presented as median ± IQR. ***p < 0.001, ****p < 0.0001.
(PDF)

S2 Fig The back arching frequency of male and female Sprague Dawley rats that received sodium pentobarbital (PB), saline controls or vehicle controls (pH 11). (a) In the female rat group, differences between saline and vehicle control groups were also observed at 151s post-injection (PI) timepoints (p < 0.05). (b) In the male rat group, no differences were observed. Data presented as median ± IQR. *p < 0.05.
(PDF)
