# Supplementary material for: Intraperitoneal injection of sodium pentobarbital has the potential to elicit pain in adult rats (Rattus norvegicus)
Source: PLoS One. 2020 Sep 3;15(9):e0238123. doi: 10.1371/journal.pone.0238123 (PMC7470368; doi:10.1371/journal.pone.0238123)
Supplement: S1 Fig — (a) In the female rat group, significant increases from baseline were observed from the vehicle control group at the 151s post-injection (PI) timepoint (p < 0.0001). Differences between saline and vehicle control groups were also observed at the 151s PI timepoint (p < 0.0001). (b) In the male rat group, differences between saline and vehicle control group were observed at the 151s PI timepoint (p < 0.001). Data presented as median ± IQR. ***p < 0.001, ****p < 0.0001. (PDF) [file pone.0238123.s001.pdf]

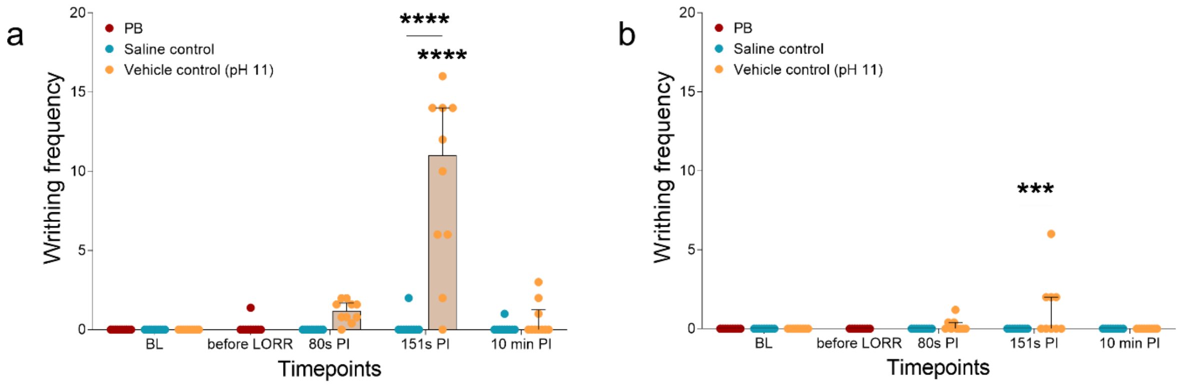

S1 Figure. The writhing frequency of male and female Sprague Dawley rats that received sodium pentobarbital (PB), saline controls or vehicle controls (pH 11). (a) In the female rat group, significant increases from baseline were observed from the vehicle control group at the 151s post-injection (PI) timepoint ($p < 0.0001$). Differences between saline and vehicle control groups were also observed at the 151s PI timepoint ($p < 0.0001$). (b) In the male rat group, differences between saline and vehicle control group were observed at the 151s PI timepoint ($p < 0.001$). Data presented as median ± IQR. ***$p < 0.001$, ****$p < 0.0001$.
