# Supplementary material for: Intraperitoneal injection of sodium pentobarbital has the potential to elicit pain in adult rats (Rattus norvegicus)
Source: PLoS One. 2020 Sep 3;15(9):e0238123. doi: 10.1371/journal.pone.0238123 (PMC7470368; doi:10.1371/journal.pone.0238123)
Supplement: S2 Fig — (a) In the female rat group, differences between saline and vehicle control groups were also observed at 151s post-injection (PI) timepoints (p < 0.05). (b) In the male rat group, no differences were observed. Data presented as median ± IQR. *p < 0.05. (PDF) [file pone.0238123.s002.pdf]

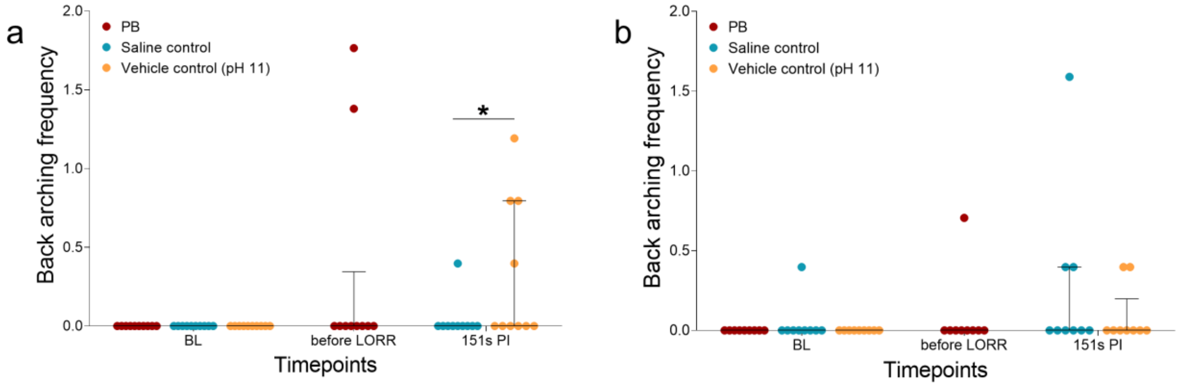

S2 Figure. The back arching frequency of male and female Sprague Dawley rats that received sodium pentobarbital (PB), saline controls or vehicle controls (pH 11). (a) In the female rat group, differences between saline and vehicle control groups were also observed at 151s post-injection (PI) timepoints ($p < 0.05$). (b) In the male rat group, no differences were observed. Data presented as median ± IQR. *$p < 0.05$.
